# Planar Tc99m – sestamibi scintimammography should be considered cautiously in the axillary evaluation of breast cancer protocols: Results of an international multicenter trial

**DOI:** 10.1186/1471-2385-5-4

**Published:** 2005-07-27

**Authors:** Teresa Massardo, Omar Alonso, Augusto Llamas-Ollier, Levin Kabasakal, Uma Ravishankar, Rossana Morales, Lucía Delgado, Ajit K Padhy

**Affiliations:** 1 Nuclear Medicine, University of Chile Clinical Hospital, Santiago, Chile; 2 Nuclear Medicine Centre and Medical Oncology Department, Hospital de Clínicas, University of La República, Montevideo, Uruguay; 3 Nuclear Medicine Department, National Cancer Institute, Bogotá, Colombia; 4 Nuclear Medicine Department, Cerrahpasa Medical Faculty, Istanbul University, Turkey; 5 Nuclear Medicine Department, Indraprastha Apollo Hospitals, New Delhi, India; 6 Department of Nuclear Medicine, Neoplastic Disease Institute and Peruvian Institute of Nuclear Energy, Lima, Peru; 7 Medicine Section, Department of Human Health, International Atomic Energy Agency, Vienna, Austria

## Abstract

**Background:**

Lymph node status is the most important prognostic indicator in breast cancer in recently diagnosed primary lesion. As a part of an interregional protocol using scintimammography with Tc99m compounds, the value of planar Tc99m sestamibi scanning for axillary lymph node evaluation is presented. Since there is a wide range of reported values, a standardized protocol of planar imaging was performed.

**Methods:**

One hundred and forty-nine female patients were included prospectively from different regions. Their mean age was 55.1 ± 11.9 years. Histological report was obtained from 2.987 excised lymph nodes from 150 axillas. An early planar chest image was obtained at 10 min in all patients and a delayed one in 95 patients, all images performed with 740–925 MBq dose of Tc99m sestamibi. Blind lecture of all axillary regions was interpreted by 2 independent observers considering any well defined focal area of increased uptake as an involved axilla. Diagnostic values, 95% confidence intervals [CI] and also likelihood ratios (LR) were calculated.

**Results:**

Node histology demonstrated tumor involvement in 546 out of 2987 lymph nodes. Sestamibi was positive in 30 axillas (25 true-positive) and negative in 120 (only 55 true-negative). The sensitivity corresponded to 27.8% [CI = 18.9–38.2] and specificity to 91.7% [81.6–97.2]. The positive and negative LR were 3.33 and 0.79, respectively. There was no difference between early and delayed images. Sensitivity was higher in patients with palpable lesions.

**Conclusion:**

This work confirmed that non tomographic Tc99m sestamibi scintimammography had a very low detection rate for axillary lymph node involvement and it should not be applied for clinical assessment of breast cancer.

## Background

Lymph node status is the most important prognostic indicator in breast cancer in recently diagnosed primary lesion. The evidence of metastatic involvement in the axilla requires the indication of adjuvant therapy posterior to surgical tumor resection. There is not an accurate anatomical test for this purpose and clinical examination has inappropriate diagnostic values. Routine lymph node dissection is the only accepted method for therapeutic decisions but it is invasive and produces significant associated morbidity such as lymphedema and, eventually, infections. On the other hand, an important proportion of breast cancer patients are node-negative. Ultrasonography has also been reported as helpful, especially adding fine needle aspiration biopsy [1,2].

The role of nuclear techniques is controversial in the area related with breast cancer [3,4]. Positron emission tomography (PET) with fluorine deoxyglucose (FDG) is an excellent method for breast cancer evaluation even though is not easily available; it is used for diagnosis and surgical planning, staging and restaging of local regional recurrence or distant metastases and also for monitoring therapy response. Its value for detecting axillary involvement is somehow debated and it has not been used in routine practice in all centers, due to its current resolution for that purpose. However, it appears to be very helpful in internal mammary node evaluation [5-8].

Sentinel node detection with radioguided biopsy has a well defined role in early staging of breast cancer and small tumors. This technique allows the recognition of lymphatic spreading. It requires nodal histology to decide complete posterior lymphadenectomy. The strategy involves diverse methodologies, is technically challenging, and requires a learning curve [9-12].

Scintimammography is widely available and its diagnostic value in axillary detection is not optimal when using planar images with 99mTc-sestamibi or phosphonates. However, reports using single photon emission tomography (SPECT) images with sestamibi and tetrofosmine labeled with Tc99m have better figures and even pinhole SPECT appears promising.

The aim of the present report was to evaluate through an unbiased standardized method the diagnostic value of planar sestamibi images for axillary involvement in breast cancer patients. This was accomplished in the scope of a multicenter interregional trial evaluating Tc99m compounds for scintimammography in breast cancer evaluation [13,14].

## Methods

### Population

This prospective study included 149 female patients ranging from 29 to 82 years (mean ± SD: 55,1 ± 11.9), from a multicenter trial on scintimammography Tc99m radiopharmaceuticals co-ordinated by the International Atomic Energy Agency (IAEA). Sixty per cent were postmenopausal. All patients had confirmed breast carcinoma (one patient had bilateral lesions). Only 50 patients (33.3%) presented also with axillary palpable nodes.

Primary breast tumour histology is documented in Table 1.

**Table 1:** Breast tumor histology

**Biopsy diagnosis**	**Number of cases**
Ductal invasive carcinoma	123
Lobular invasive carcinoma	14
Colloidal carcinoma	5
Tubular carcinoma	3
Carcinoma *in situ*	3
Medullary carcinoma	1
Sarcoma	1
**Total**	**150**

The median size breast lesion was 25 mm ranging from 7 – 80 mm (mean ± SD = 28.8 ± 13.9 mm).

Scintimammography was performed before the histopathological confirmation of the cancer. Cases with fine-needle aspiration as the only confirmatory procedure were excluded. Axillary lymph node dissection in 150 axillary beds was performed as a part of the standard staging.

All patients included in this group provided written informed consent according to their local institutions at participating centres (Chile, China, Colombia, Cuba, Greece, India, Peru, Turkey and Uruguay).

### Tc-99m scintimammography protocol

The same protocol was used in all centres. The radiochemical purity of Tc-99m-MIBI was ≥95%. Patients were injected with a bolus of 740 -925 MBq of sestamibi into an antecubital vein in the contra-lateral arm to the breast lesion or in a pedal vein in the patient with bilateral lesions. A plastic cannula was used to avoid interstitial infiltration and the injection was followed by a saline flush.

The acquisition began 10 min post injection with the patient supine. Imaging parameters were: matrix 256 × 256, peak energy of 140 ± 10% KeV, high-resolution low-energy collimator. The breast-collimator distance was kept to a minimum and a static 10 min image was always acquired. Anterior thoracic images included the neck, both axillas and breasts (with arms up). Lateral views were obtained with the patients in prone position using a commercially available breast pad set, (Pinestar Technology, Inc. Greenville, PA, USA), allowing the organ to hang freely, compressing the contra lateral breast. Delayed images were also obtained 90 min post injection in 95 patients using the same protocol. The gamma cameras were standard for clinical practice, including GE Starcam o, Elscint Apex, Siemens Diacam, and Sopha Sophy. Standardized contrasted images in gray scale were recorded.

### Data analysis

All scintimammograms were interpreted by two experienced nuclear medicine physicians, blinded to clinical status of the patients as well as to all other tests results. The readers decided if the scan was positive or negative for lymph node involvement in both axillas. One or more focal areas of increased sestamibi uptake was considered positive. Their number was also consigned. The injection site was available for the observers only when a false positive interpretation was suspected due to radiopharmaceutical retention in a lymph or venous vessel.

Lymph node histology was considered as the gold standard. Results were incorporated to *Arcus Quickstat *and *Instat *data set for analysis.

Diagnostic values with a 95% confidence interval [CI] and Likelihood Ratios (LR) were calculated. Student t test was applied.

## Results

One-hundred and fifty axillary lymph node dissections were performed in the 149 patients. Malignant involvement was reported in 89 out of 149 patients, (90 axillas). A total of 2987 lymph nodes were removed with a range of 4–47 nodes per patient (mean ± SD: 19.9 ± 9.7). Of these 2987 nodes, 546 presented histological tumoral status.

Sestamibi scintimammography was positive in 30 axillas (25 of them true-positive) and negative in 120 (55 true-negative). Thus, the sensitivity corresponded to 27.8% [CI = 18.9–38.2] and specificity to 91.7% [CI = 81.6–97.2]. The positive and negative LR were 3.33 and 0.79, respectively.

Two thirds of the axillas with single node involvement were false-negative (12 cases). When multi-nodal involvement was present, 31 cases with 2–5 nodes were false negative as well as 14 cases with 6–10 nodes, and in cases with more than 11 nodes involved, 8 cases were false-negative. There was a trend to lower sensitivity in the axillas with less than 5 nodes involved: 13.8 % versus 32.4% (p:0.47). With the currently used cut-off of 3 nodes involved, 53% of the false-negatives axillas were equal or under that number.

The only five false-positives corresponded to reactive lymphadenitis, follicular hyperplasia or were just specified as non-malignant.

**Figure 1:**
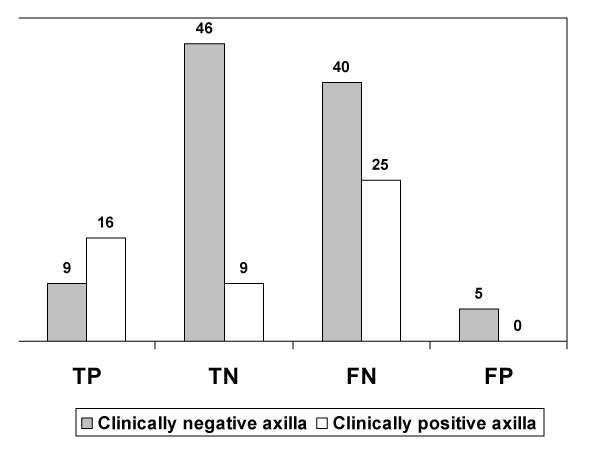
Tc99m sestamibi performance according to axillary status.

The sensitivity of scintimammography in the group with palpable axillary nodes was significantly higher than in the non palpable group (p:0.036). They corresponded to 39.0% [CI = 8.8–32] versus 18.4% [CI = 24.2–55.5]. Specificities were 100% [CI = 66.4–100] versus 90,2% [CI = 78.6–92.7]; positive LR was 3.9 versus 1.87 and negative LR 0.61 versus 0.91, respectively. See Figure 1.

**Figure 2:**
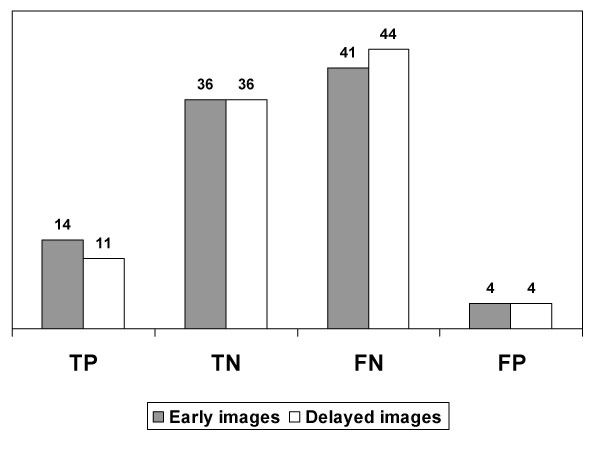
Comparison between axillary results in early and delayed Tc99m sestamibi in 95 patients.

There was no difference between early and delayed diagnostic values in the 95 patients with both exams performed in identical conditions (p:0.65). See Figure 2.

## Discussion

These results support that planar imaging with scintimammography and Tc99m- sestamibi should be definitively excluded or considered cautiously for axillary evaluation protocols in breast cancer.

### Different techniques for axillary evaluation

Yutani et al. [15] in their comparative study between FDG PET and sestamibi-SPECT reported sensitivities of 50.0 and 37.5%, respectively, for axillary detection in 40 consecutive patients with head to head comparison. Their results with tomographic images are relatively concordant with ours. However, in this setting, theirs and our sensitivity values were disappointingly low and are clearly opposed to several prior reports with either planar or SPECT techniques (See Table 2; [15-28]). This could be explained by the size and depth of the lesions, their relative low uptake and especially by the equipment resolution. Our lower detection rate compared with other reports may be explained, in part, by the method of robust blind reading with no interpretation bias.

**Table 2:** Scintimammography results according to number of axillary nodes involved

**N° involved nodes/axilla**	**N° axillas False-Negative**	**N° axillas True-Positive**
1	12	6
2–5	31	*
6–10	14	*
11–20	4	*
>20	4	*
Total	65	25

* Individual group data is not available (2 nodes or more subgroups = 19 nodes)

It is interesting to mention that sestamibi is helpful for the diagnosis of melanoma lymph node assessment [29], contrary to the observed situation discussed in breast cancer. The reason for this fact could be the most superficial and somehow easier to locate melanomatous involved nodes. The nodes in axillas are deeply positioned which can probably contribute to the lower sestamibi uptake in breast cancer.

PET FDG has been proposed in order to reduce the proportion of patients requiring axillary dissection with variable results, but until now the technique cannot adequately assess the number of nodes involved. However, it could be very helpful in the evaluation of internal mammary chain in upper medial quadrant primary tumours, as well as in patients with large lesions. According to Danforth et al. [30] in 495 patients its global sensitivity for axillary involvement was 89% [95%CI = 86–92], with a specificity of 87% [95%CI = 84–90]. Yutani et al. [15] reported that FDG is sufficiently sensitive to rule out lymph node metastasis. Greco et al. [5] reported in 167 patients FDG sensitivity of 94%, specificity 86% and accuracy of 90% for axillary evaluation.

We agree with other authors [15,23] who have published that planar scintimammography is not recommended for axillary evaluation. Tolmos et al. [20] do not consider the test as reliable (they observed a kappa value of 0.49 for interobserver agreement). Even though, there are posterior and recent publications with new results still reporting relatively good values [17,25-28]. Limachi et al. [27] reported lower sensitivity if fewer nodes were affected, similar to our findings (in patients with <3 metastases, sensitivity was 69.7%, and only one out of six patients with a single lesion had a positive scan). See Table 3.

**Table 3:** Diagnostic value of the published literature (PUBMED) in breast cancer axillary lymph node evaluation using Tc99m sestamibi.

**Author**	**Sensitivity (%)**	**Specificity (%)**	**N° of patients**	**Ref.N°**
Lam et al. *Eur J Nucl Med, 1996*	64	90	31	16
Cistaro et al. *Minerva Chir*, 1997	75	90	45	17
Schillaci et al. *Anticancer Res, 1997*	61.9 81 *	96.4 92.9 *	49	18
Akcay et al. *Clin Nucl Med, 1997*	66	100	30	19
Tolmos et al. *Am Surg, 1997*	75	82	31	20
Perre et al. *Eur J Surg Oncol, 1997*	91	64	36	21
Taillefer et al. *J Nucl Med, 1998*	79.2	84.6	100	22
Danielsson et al. *Acta Radiol, 1999*	67	80	58	23
Arslan et al. *Nucl Med Commun, 1999*	68	93	77	24
Mulero et al. *Rev Esp Med Nucl, 2000*	36	100	84	25
Yutani et al. *J Comput Assist Tomography, 2000*	38*	NA	40	15
Nishiyama et al. *Eur J Nucl Med, 2001*	73	NA	50	26
Lumachi et al. *Eur J Surg Oncol, 2001*	82.3	94.1	239	27
Chen et al. *Chin Med J, 2003*	83.3	86.1	60	28
***IAEA group***	***28***	***92***	***149***	

NA : Not available

* : SPECT

### Other compounds labeled with Tc99m

Regarding data with other compounds labeled with Tc99m, commonly used, especially tetrofosmin also a cationic lipophilic molecule, the values are similar to sestamibi in breast cancer evaluation [19,31]. Akcay [19] found comparable diagnostic value for both in a small number of patients with involved axillary nodes. The experience with SPECT is significantly better including small primary breast tumours [32]. Tc99m diphosphonates (MDP) proposed as an interesting alternative as well as pentavalent DMSA, have less diagnostic value than sestamibi for breast primary lesions and also for axillary node evaluation, according to our group results and others [13,26].

### The addition of P-SPECT

Madeddu and Spanu, using tetrofosmin, proposed recently SPECT with pinhole (P-SPECT) as the best technique to evaluate the axilla. Their group demonstrated that P-SPECT has better sensitivity compared to SPECT and they, individually, were superior to planar imaging, even for non palpable axillary lesions [33-35]. Their group previously reported also that tetrofosmin SPECT has better sensitivity than planar scintimammography for palpable and non palpable axillary lesions [36]. When P-SPECT was performed with sentinel node detection both techniques combined gave 100% accuracy and P-SPECT was able to identify 81.2% of cases with a single node, and correctly classified 93.7% of the patients with ≤ or > 3 metastatic nodes [37].

### Other interesting points

It has been reported that sestamibi and FDG are related with low radiopharmaceutical uptake in early forms of breast carcinoma that make tumoral detection more difficult in certain cancer subtypes, such as invasive lobular carcinoma and low-grade tumors, even with locally advanced disease [38-40]. It appears that favorable response to neoadjuvant therapy, in locally advanced disease is complex due to tumoral flow and metabolic changes [41].

Finally, it should be considered that in women with a clinically negative axilla the information obtained from surgical dissection in order to decide adjuvant therapy is related to age and other factors, such as tumor characteristics [42]. SPECT equipment capacity should be ameliorated in order to improve the detection of smaller lesions in breast carcinoma, as was published with phantom models [43]. The recent and excellent review by Taillefer (44) regarding scintimammography suggested that it is necessary to define the clinical niches of the test. In axilla, the diagnostic accuracy of sestamibi varied between 80–85% (with an overall accuracy of 81% (411/509) for 12 reports including two with SPECT); for him, this value is still too low to advocate its use to avoid axillary node dissection in patients with proven invasive primary breast cancer.

## Conclusion

There is strong information supporting that planar sestamibi data is not an adequate alternative for axillary evaluation in breast cancer. We believe that countries with limited resources regarding radiopharmaceuticals and equipment availability, should avoid the non-tomographic protocol.

## List of abbreviations

CI: Confidence Interval

LR: Likelihood Ratio

PET: Positron Emission Tomography

FDG: Fluorine deoxyglucose-F18

SPECT: Single Photon Emission Tomography

P-SPECT: SPECT with pinhole

## Competing interests

The author(s) declare that they have no competing interests.

## Authors' contributions

TM carried out nuclear studies and participated in the design and discussion, read blindly all the studies and redacted the final manuscript

OA carried out nuclear studies and participated in the design and discussion, read blindly all the studies performed the statistical analysis and reviewed the final manuscript

AL-0 carried out part of the studies and participate in the former redaction of the manuscript

LK carried out nuclear studies and participated in the design and discussion

UR carried out nuclear studies and participated in the design and discussion

RM carried out nuclear studies and participated in the design and discussion

LD analyzed the oncological data

AKP, as the chief of the IAEA research group, conceived the study, participated in the design and global coordination.

All authors read and approved the final manuscript.

## Pre-publication history

The pre-publication history for this paper can be accessed here:


